# Nanostructured Mn@NiO composite for addressing multi-pollutant challenges in petroleum-contaminated water

**DOI:** 10.1007/s11356-024-34012-3

**Published:** 2024-06-28

**Authors:** Gamil Gamal Hasan, Salah Eddine Laouini, Ahmed I. Osman, Abderrhmane Bouafia, Mohammed Althamthami, Souhaila Meneceur, Iman Kir, Hamdi Mohammed, Brock Lumbers, David W. Rooney

**Affiliations:** 1 Laboratory of Valorisation and Technology of Sahara Resources (VTRS), El Oued University, 39000 El Oued, Algeria; 2 Laboratory of Biotechnology Biomaterials and Condensed Matter, Faculty of Technology, University of El Oued, 39000 El Oued, Algeria; 3 School of Chemistry and Chemical Engineering, Queen’s University Belfast, David Keir Building, Stranmillis Road, Belfast, Northern Ireland BT9 5AG UK; 4 Physics Laboratory of Thin Films and Applications, Biskra University, BP 145, 07000 Biskra, RP Algeria; 5 Faculty of Technology and Bionics, Rhine-Waal University of Applied Sciences, Marie-Curie-Straße 1, 47533 Kleve, Germany

**Keywords:** Green synthesized, Petroleum water treatment, Degradation mechanism, Nanocomposite, Organic contaminants, Pharmaceutical product

## Abstract

Efficient catalysts play a pivotal role in advancing eco-friendly water treatment strategies, particularly in the removal of diverse organic contaminants found in water-petroleum sources. This study addresses the multifaceted challenges posed by contaminants, encompassing a spectrum of heavy metals such as As, Cd, Cr, Mn, Mo, Ni, Pb, Sb, Se, and Zn alongside pollutants like oily water (OIW), total suspended solids (TSS), chemical oxygen demand (COD), dyes, and pharmaceuticals, posing threats to both aquatic and terrestrial ecosystems. Herein, we present the synthesis of biogenically derived Mn@NiO nanocomposite (NC) photocatalysts, a sustainable methodology employing an aqueous Rosmarinus officinalis L. extract, yielding particles with a size of 36.7 nm. The catalyst demonstrates exceptional efficacy in removing heavy metals, achieving rates exceeding 99–100% within 30 min, alongside notable removal efficiencies for OIW (98%), TSS (87%), and COD (98%). Furthermore, our photodegradation experiments showed remarkable efficiencies, with 94% degradation for Rose Bengal (RB) and 96% for methylene blue (MB) within 120 min. The degradation kinetics adhere to pseudo-first-order behavior, with rate constants of 0.0227 min^−1^ for RB and 0.0370 min^−1^ for MB. Additionally, the NC exhibits significant antibiotic degradation rates of 97% for cephalexin (CEX) and 96% for amoxicillin (AMOX). The enhanced photocatalytic performance is attributed to the synergistic interplay between the Mn and NiO nanostructures, augmenting responsiveness to sunlight while mitigating electron-hole pair recombination. Notably, the catalyst demonstrates outstanding stability and reusability across multiple cycles, maintaining its stable nanostructure without compromise.

**Graphical Abstract:**

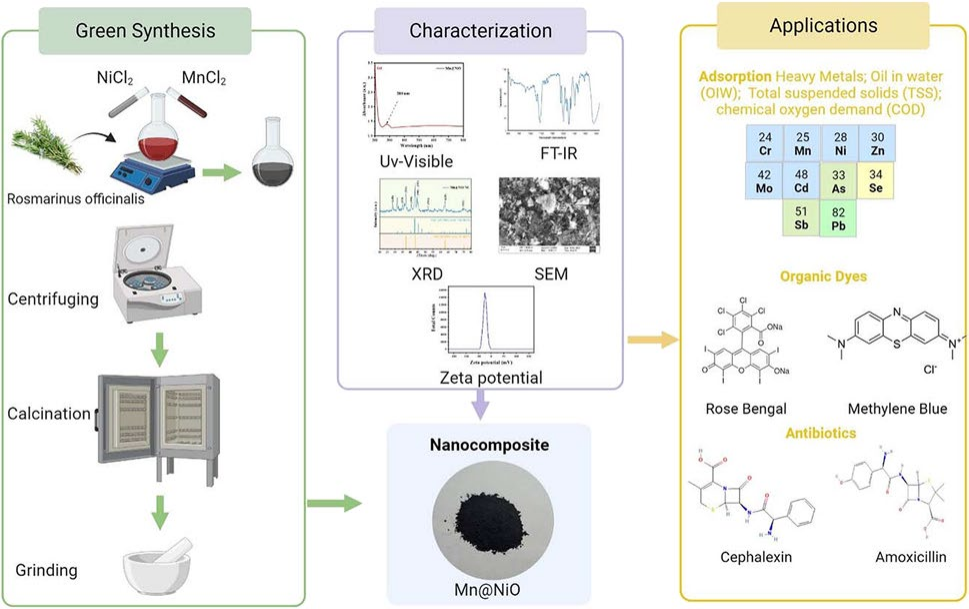

**Supplementary Information:**

The online version contains supplementary material available at 10.1007/s11356-024-34012-3.

## Introduction: Turner designs hydrocarbon instrument

The growing concern surrounding the reduction of environmental damage caused by waste production, encompassing water, soil, and air pollution, has spurred research into innovative approaches to enhance traditional environmental treatment methods (Bender et al. 2019; Sibhatu et al. 2022). Advanced technologies have emerged, refining and optimizing established remediation practices. One significant advancement attracting attention is the synthesis and precise engineering of nanoscale metallic particles (Hasan et al. 2023a), This allows customization of their shapes, sizes, and compositions (Harish et al. 2022; Rathinabala et al. 2022). These nanomaterials have found diverse applications in energy, electronics, optoelectronics, magnetics, cosmetics, biomedicine, pharmaceuticals, and catalysis (Arora et al. 2022; Hariharan et al. 2020; Sagadevan et al. 2023; Shafey 2020). Particularly, the unique properties of nanoparticles (NPs), intricately tied to their shapes and sizes, highlight their versatile uses (Mohammed Mohammed et al. 2023). In this context, metal oxide nanoparticles (MO NPs) have emerged as promising catalysts, capitalizing on their exceptional and multifaceted properties (Suppiah et al. 2023). The catalytic capabilities of MO NPs have attracted significant interest due to their ability to facilitate intricate chemical reactions. Of particular note is their pivotal role in eliminating pollutants, specifically in remediating environmental contaminants in air, water, and soil matrices (Osman et al. 2023). Recent studies have highlighted the impressive effectiveness of metal oxide nanoparticles in breaking down colored dyes (Kumari and Pandey 2023; Yaghoobi et al. 2023).

Wastewater treatment is a crucial process aimed at eliminating pollutants and various contaminants from wastewater before its release into the environment or potential reuse (Balakrishnan and Chinthala 2023). However, traditional methods of wastewater treatment have room for improvement in removing specific pollutants (Tawfik et al. 2022).

Nanotechnology has emerged as a vital tool, allowing the manipulation of matter at the atomic, molecular, or nanoscale levels. This unique capability positions nanotechnology to significantly enhance the efficiency and effectiveness of wastewater treatment processes (Kumar et al. 2022b; Singh et al. 2023). Consequently, a burgeoning interest has emerged in the application of nanotechnology within wastewater treatment domains (Qu et al. 2013). As a result, there is growing interest in applying nanotechnology in wastewater treatment, showing promise in developing nanoparticles capable of adsorbing or removing pollutants from wastewater (Althamthami et al. 2022; Kumar et al. 2022a). Certainly, ZnO@CuO NPs have proven to be remarkably efficient in removing various pollutants, such as heavy metals (Meneceur et al. 2023). Additionally, Fe_3_O_4_@SnO_2_ NC has demonstrated its catalytic prowess in degrading substances such as methylene blue, Rose Bangel, amoxicillin, and cephalexin (Hasan et al. 2023b).

The escalation of heavy metal pollution, a problem dating back to the 1940s and persisting today, is chiefly attributed to rapid urbanization and industrialization (Ali et al. 2019). Emerging nations witness increased discharge of heavy metals from sectors like refineries, petrochemicals, and manufacturing, posing a significant challenge to society (Khan et al. 2004). Heavy metals, like mercury and lead, persist in the environment and can enter the human food chain, causing toxicity and health issues (Merian 1984). Current water treatment methods, though effective, generate secondary waste and come at a high cost (Huang et al. 2014). This study aims to tackle these issues by developing eco-friendly methods to produce nanoscale particles inexpensively and efficiently. These particles can eliminate various types of waste, offering a sustainable solution to heavy metal pollution. The objective is to overcome the limitations of existing methods, ensuring the removal of contaminants while promoting environmental sustainability.

Consequently, scientists are exploring alternative methods, such as nanotechnology, to mitigate this issue (Jharwal et al. 2022). Specifically, researchers have investigated the efficacy of manganese (Mn) and nickel oxide (NiO) nanoparticles (NPs) in dye removal, owing to their durability and non-toxic nature (Al Boukhari et al. 2019). These NPs are attractive for their durability and non-toxicity (Dessie et al. 2021). The study of Mn and NiO NPs not only offers a promising solution for wastewater treatment but also lays the groundwork for more potent and sustainable approaches in this field (Liu et al. 2022).

The MnO_2_ and NiO nanoparticles (NPs) have also been studied for their potential in removing antibiotics such as cefalexin from wastewater; the research findings underscore a notable achievement, revealing an impressive degradation efficiency of 99.06% for cefalexin. Therefore, nanotechnology, specifically Mn and NiO NPs, has been explored as a potential method for removing these antibiotics from wastewater (Alarfaj et al. 2021).

Methylene blue (MB) and Rose Bengal (RB) are commonly used dyes in various industries and can have harmful effects on the environment when released into wastewater due to their toxicity and persistence (Hassan et al. 2021; Mishra et al. 2023).

Vilardi et al. (Vilardi et al. 2018) conducted an extensive examination of the utilization and impact of metal oxide nanoparticles in biological wastewater treatment, focusing on methods to assess nitrification inhibition. Another study, Yang et al. (Yang et al. 2013) explored the fate and consequences of various nanoparticles in wastewater and anaerobic digestion. Specifically, Sing et al. (Singh et al. 2019) specifically investigated the effects of TiO_2_ and iron oxide nanoparticles in the context of wastewater treatment; the findings related to TiO_2_ revealed a discernible adsorption capacity of 5.18 mg/g for Cu(II) and a significantly elevated adsorption capacity of 61.07 mg/g for Fe_3_O_4_ NPs. Furthermore, Junbai et al. (Fei and Li 2007) provided an important insights into metal oxide materials and associated techniques for treating different water resources affected by organic and inorganic anions.

The use of Mn and NiO nanoparticles (NPs) to remove pollutants from wastewater is an exciting avenue for addressing the problem of pollution in the environment. However, more research is needed to evaluate the effectiveness of these NPs in eliminating different dyes and antibiotic classes, as well as any potential negative impacts on the environment (Manigandan et al. 2023).

In this investigation, we synthesized a Mn@NiO NC using an environmentally conscious approach that tapped into the efficacy of a Rosmarinus officinalis L. extract as a sustainable reducing agent. This groundbreaking research signifies the maiden use of Rosmarinus officinalis L. extract in crafting Mn@NiO NC tailored for the simultaneous removal of organic contaminants in petroleum water, as well as the extraction of organic dyes and pharmaceutical products from wastewater. Furthermore, our study is distinctly focused on tackling the complex challenge of segregating hydrocarbons, suspended solids, and heavy metals inherent in petroleum wastewater. This intricate separation process is accomplished by harnessing NC derived from vegetable waste for efficient adsorption purposes.

## Experimental section

### Reagents and material characterization

*Rosmarinus officinalis L*., a plant from Algeria’s El Oued area, served as the study’s botanical source. The chemical agents used in the experiment included acetone (C_3_H_5_O), methylene blue (C_37_H_27_N_3_O_9_S_3_Na_2_), and Rose Bengal (C_20_H_2_Cl_4_Na_2_O_5_), which were acquired from Biochem Chemopharma. Manganese (II) chloride (MnCl_2_ 98%) and Nickel (II) chloride (NiCl_2_) were purchased from Sigma-Aldrich.

### Preparation of *Rosmarinus officinalis* L. extract and green synthesis of Mn@NiO NC

To prepare a *Rosmarinus officinalis L*. extract solution was sourced from a local botanical garden in El Oued, Algeria. The leaves were cleaned with deionized water (DW), dried, and crushed finely. The resulting 100 g of crushed leaves were then boiled in 500 ml of deionized water at a concentration of 100 g/500 mL, heated to 70 °C for 2 h, and vacuum-filtered before being stored at 4 °C for further use. MnCl_2_, obtained from Sigma-Aldrich, was used as a precursor for synthesizing Mn NPs. The rosemary mentioned above extract was added to 0.1 M MnCl_2_ at a 1:1 volume ratio and left to incubate at ambient temperature. Next, NiCl_2_ was introduced into the solution to synthesize Mn@NiO NC. After centrifuging at 3000 rpm for 10 min, the Mn@NiO NC solution was washed with acetone and DW with the centrifuging process and then dried at 70 °C for 8 h to ensure that all residual solvent was completely evaporated.

### Characterization of the biosynthesized Mn@NiO NC

The present study focused on analyzing the optical properties of Mn@NiO NC that were synthesized through a biological process. To determine the optical characteristics of the NC, a UV-visible spectrophotometer (Shimadzu UV-2450, USA) was used to record absorbance spectra in the wavelength range of 200–900 nm. The functional groups present in the synthesized NC were also identified using FTIR in diffuse reflection mode in the range of 4000–400 cm^−1^. X-ray diffraction (XRD) study was carried out using a Shimadzu XRD-7000 diffractometer within the 2θ range of 25–90°, and the crystallite size was calculated using the Scherrer equation to evaluate the crystallinity and grain size of the Mn@NiO. Finally, the morphology of the biosynthesized Mn@NiO NC was evaluated using SEM.

### Adsorption study of metal ions on Mn@NiO NC

Sorption experiments were conducted on Mn@NiO NC in the presence of ten different metal ions (As, Cd, Cr, Mn, Mo, Ni, Pb, Sb, Se, and Zn). The metal ions were in the form of nitrates to ensure consistent effects of the counter ions. Each experiment combined 20 mg of NC with 10 mL of oily water. The mixture was then supplemented with solutions containing varying concentrations of the metal ions. Subsequently, the resulting mixture underwent 30 min of sonication and was separated using a magnet. The concentration of metal ions in the liquid remaining above the solid was determined using inductively coupled plasma mass spectrometry (ICP-MS) analysis, specifically employing the ICP-MS Model NexION 2000 from the USA. The results were analyzed using the Syngetix operating software integrated into the ICP device. The metal ion concentrations were measured in mg/L through linear regression analysis, utilizing the response of the standards. The obtained values were adjusted with the assistance of internal standards. If necessary, the data were reanalyzed using appropriate software, and in cases where dilution was involved, the values were multiplied by the respective dilution factor.

### Oily in water sample (OIW) analysis

To calibrate the TD-500 meter, a hydrocarbon oil in water analyzer manufactured by Turner Designs Hydrocarbon Instruments (USA), a standardized sample is employed. Initially, predetermined standard samples containing a specific oil content are prepared. A bottle is filled with 100 mL of water, and its pH is adjusted to below 2 by adding approximately 4 or 5 drops of HCl. Then, 10 mL of hexane solvent is added to the sample, and the mixture is vigorously shaken for 2 min to extract hydrocarbons from the water. After allowing the mixture to settle for approximately 10 min, around three-fourths of the liquid from the test socket in the ampoule is extracted using a syringe. Prior to placing it in the device’s bowl, the test socket is carefully cleaned. The analyzer provides a response value, typically within 5 s, which is then read. The concentration of hydrocarbons, measured in parts per million (ppm), is recorded for further analysis purposes.

### Total suspended solids (TSS) preparation

In this procedure, a precise volume of 10 mL from a water sample containing petroleum was carefully transferred into a transparent vessel, guaranteeing unobstructed light transmission through the sample. The vessel containing the prepared sample was then inserted into a UV-visible spectrophotometer, and the analysis was initiated. Following a period of 10 min, the resulting measurement was observed and displayed on the instrument’s screen.

### Equipment used for adsorption study of metal ions

Inductively coupled plasma mass spectrometry (ICP-MS) is an extremely sensitive method used to measure the proportions of metals and distinguish various ions within the same solution. The ICP-MS product HTDS, Model NexION 2000, comprises components such as a spray chamber, nebulizer, valve system, and operating software. Additionally, it necessitates specific supplies like gas cylinders, ultra-pure water, and standard solutions. The adsorbents used were particles of Mn@NiO NC synthesized through an environmentally friendly method.

To quantify the amount of oil or oil-in-water (OIW) in the separated or generated water, an oil-in-water analyzer (TD-500, Turner Designs Hydrocarbon Instruments, USA) was employed. This device, identified by revision C and part number 100,668, was sourced from the USA. It utilizes UV fluorescence technology to determine the oil content in oily water, including crude oil or gas condensates.

### Photocatalytic performance of organic pollutants

To assess the photocatalytic efficacy of Mn@NiO NC in degrading methylene blue (MB) and Rose Bengal (RB) dyes under sunlight irradiation, initially, dye solutions were made by dissolving 10 ppm/L of every dye and mixing them with Mn@NiO NC at consistent intervals. After equilibrating the solution for 5 min without light, each sample consisting of 5 mg of NC and 5 mL of dye was analyzed at (0, 15, 30, 45, 60, 90, and 120 min). Following centrifugation of the solution prepared at 3000 rpm for 5 min to separate the Mn@NiO NC from the organic dye solution, the supernatant was analyzed using UV-visible spectrophotometry between 400 and 800 nm to evaluate the degradation of the dye (Hasan et al. 2023c).

### Photocatalytic activity of antibiotics

To evaluate the antibiotics adsorption efficiency of Mn@NiO NC, a solution of 50 mg cephalexin (CEX) and amoxicillin (AMOX) in 50 mL of distilled water was added to a 50-mL colorimetric glass tube at pH 7.2, containing a 25 mg of Mn@NiO NC. The mixture was agitated on a rotary shaker at 300 rpm for fixed intervals of 10, 20, 30, 40, and 50 min; the samples prepared were exposed to a 420-nm ultraviolet filter (Xenon lamp at 300-watt ). The (CEX, AMOX) were then measured in solution using a UV-visible spectrophotometer, and the adsorption efficiency (D%) of (CEX, AMOX) by Mn@NiO NC was calculated using Eq. (1), where *C*_0_ and *C*_*t*_ are the concentrations of (CEX, AMOX) at time zero and time *t*, respectively. This procedure was employed to evaluate the adsorption performance of the synthesized Mn@NiO NC.

1 $$D\left(\%\right)=\left({C}_0-{C}_t/{C}_0\right)\times 100$$

## Results and discussion

### Optical characteristics and bandgap

**Fig. 1:**
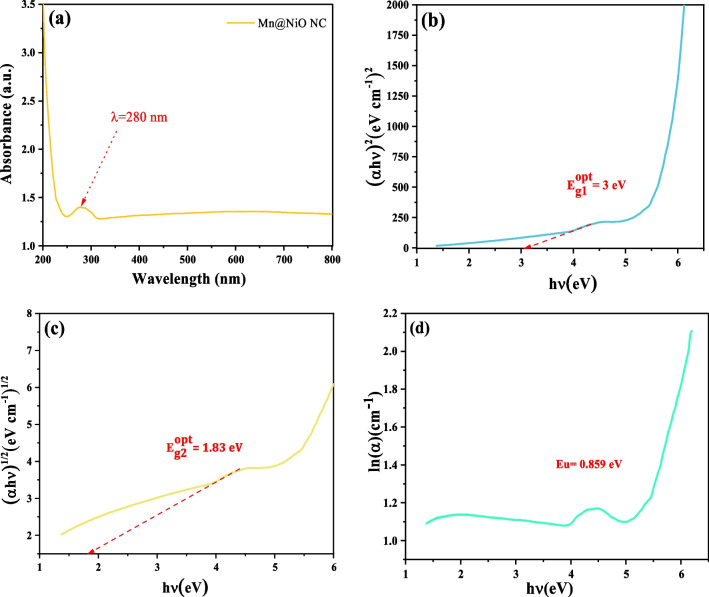
Optical properties of the Mn@NiO N. UV-Vis spectra (**a**), optical energy bandgap (**b**), indirect energy bandgap (**c**) transitions relying on Tauc’s technique, and Urbach energy (**d**)

The Mn@NiO NC UV-Vis absorption spectra are displayed in Fig. 1a. The visible spectrum absorption of Mn@NiO NC is shown in Fig. 1a as *λ*_max_ = 280 nm. The Mn@NiO NC using *Rosmarinus officinalis L.* exhibits UV-Vis absorption spectra with a visible spectrum absorption of *λ*_max_ = 280 nm, showing that this NC strongly absorbs light in the UV region. This absorption behavior can be attributed to the presence of both Mn and NiO, which exhibit pronounced UV absorption characteristics (Mala et al. 2023). The absorption spectra of compounds provide insights into their electronic structure, including the energy levels of electrons and the bonding interactions between atoms. In this context, the absorption peak observed at 280 nm signifies a substantial degree of electron delocalization within the compound, a trait commonly associated with metals and metal oxides (Guo et al. 2022).

The Mn@NiO NC’s usage of *Rosmarinus officinalis L.* as a stabilizing and reducing agent may also impact the absorption spectra. The natural antioxidant *Rosmarinus officinalis L.* is rich in phenolic compounds, which might interact with the metal ions in the NC and alter their electronic structure (Farshchi et al. 2018).

Tauc’s formula was used to estimate the band gap energy of the Mn@NiO NC by plotting the coefficient of absorption versus photon energy. When estimating the band gap energy of semiconductor materials, scientists commonly used Tauc’s formula. According to the following formula, the absorption coefficient (α) is related to the photon energy (hv) and the band gap energy (Eg) (Eq 2) (Tauc and Menth 1972):

2 $$\left(\alpha hv\right)=A{\left( hv-{E}_g^{opt}\right)}^n$$

where *α* is the absorption coefficient, *hv* is the energy of the photons that were incident, ${E}_g^{opt}$ is the band gap energy in electron volts (eV), *K* is a constant, and *n* is the power factor that depends on the type of electronic transition.

Generally, *n* is 2 for direct transitions and 1/2 for indirect transitions, as shown in Fig 1(b, c) and Table S1. The absorption coefficient (*α*) is multiplied by the square root of the photon energy, ${\left(\alpha hv\right)}^2\ \textrm{and}{\left(\alpha hv\right)}^{\frac{1}{2}}$, and the result is plotted as a function of photon energy to get the band gap energy. The band gap energy ${E}_{g1}^{opt}$ for direct transitions may be calculated from the *x*-intercept of the linear fit, whereas the band gap energy ${E}_{g2}^{opt}$ for indirect transitions can be calculated from the point where the linear fit intersects the photon energy axis (Legmairi et al. 2023; Zidane et al. 2022).

The linear portion of the (ln *α*) vs photon energy slope may be used for estimating the Urbach energy; this is a measure of the band tails width in localized states, as depicted in Fig. 1(d). The Urbach energy (Eu) is the energy required to excite an electron from the valence band to the tail of the density of states in the conduction band, and it is connected to the disorder in the material. It is expressed as (Eq. 3):

3 $$\alpha (E)={\alpha}_0{\exp}^{\left(E-{E}_g/ Eu\right)}$$

where *E* is the photon energy, *E*_*g*_ is the energy bandgap, and *Eu* is the Urbach energy. *α*(*E*) is the coefficient of absorption at energy *E*. *α*_0_ is a constant. The Urbach energy *Eu*, which may be estimated using the formula below (Eq. 5), corresponds to the slope of the linear portion of the (ln *α*) vs photon energy plot.

4 $$Eu=\left(h/\alpha \right)\times \left( d\alpha / dE\right)$$

where $\frac{d\alpha}{d E}$ is the linear portion slope of the (ln *α*) versus photon energy plot, and *h* is Planck’s constant. *α* is the coefficient of absorption. Urbach energy may provide information on the disorder and quality of the material (Table 1). The performance of electronic devices can be impacted by greater localized state density and a higher degree of disorder, both of which are indicated by greater Urbach energies (Galvani et al. 2019).

**Table 1:** The different energy values of Mn@NiO NC prepared using *Rosmarinus officinalis L*. extract

Samples	Direct optical bandgap (eV)	Indirect optical bandgap (eV)	Urbach energy (eV)
Mn@NiO NC	3	1.83	0.859

### X-ray diffraction analysis

**Fig. 2:**
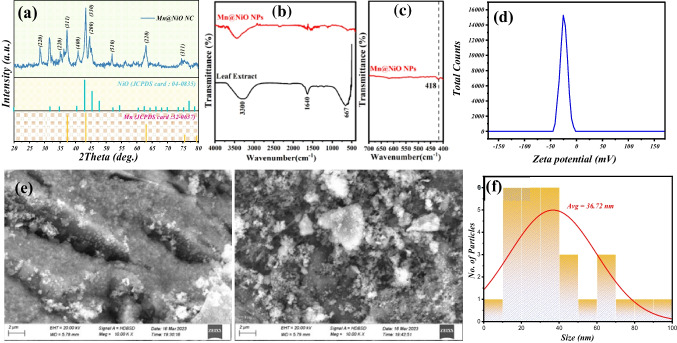
The Mn@NiO NC characterization. **a** XRD patterns. **b**, **c** FT-IR analysis. **d** Zeta potential. **e** SEM image. **f** Average particle size distribution

The X-ray diffraction (XRD) illustrated in Fig. 2a displays the Mn@NiO NC that was synthesized using *Rosmarinus officinalis* extract leaves. The XRD pattern revealed distinct diffraction peaks located at 37°, 44°, 62°, 75°, and 79° that corresponded to the (111), (200), (220), (311), and (222) crystal planes, respectively. These peaks demonstrated the cubic phase structure of NiO. The observed diffraction peaks were in perfect agreement with the standard card (JCPDS card no: 04-0835), which validated the formation of NiO NPs (Sankar et al. 2016). The peaks located at 24°, 28°, 31°, 40°, 45°, and 52° are corresponding metallic manganese sources, indicating that it is pure manganese, as the JCPDS reference card: 32-0637; it has been observed that certain peaks have undergone slight shifts. The crystallite size of the Mn@NiO NC was calculated for position 2θ=43.26° using the Debye-Scherrer equation (Eq. 5), where the *D* is the crystallite size estimated to be 19 nm.

5 $$D=0.9\lambda /\beta \cos \theta$$

The XRD pattern shows no additional peaks, indicating that the transition metal ions were incorporated into the Ni sites without altering the cubic structure. The reduction in diffraction peak intensity suggested a loss of crystallinity caused by lattice distortion due to incorporating Mn concentration into the NiO lattice. Mn@NiO NC exhibited lower diffraction peak intensity than undoped NiO NPs due to Mn ions being introduced into the periodic lattice of NiO, which induced strain at the Ni sites. This strain altered the lattice periodicity and decreased the crystal symmetry of Mn@NiO nanoparticles (Gopinadhan et al. 2007). It is possible that additional factors, such as modifications to the particle’s size, shape, or flaws, contributed to the decrease in diffraction peak intensity rather than just the addition of Mn to the NiO lattice. It is also likely that in addition to Mn incorporation, a combination of these factors contributed to the decrease in peak intensity.

### FTIR spectroscopy analysis

The functional groups of organic molecules may be identified via FT-IR analysis, which can also be utilized to discover possible phytochemical compounds that are involved in the green synthesis of the NC. These phytochemicals could consist of flavonoids, terpenoids, and phenolic compounds, which are known as reducing and stabilizing agents that might help in the synthesis of nanoparticles. The synthesized powder of Mn@NiO NC and the leaf extract of *Rosmarinus officinalis L*. were both subjected to FT-IR analysis in this case. The *Rosmarinus officinalis L.* leaf extract’s FT-IR spectra, depicted in Fig. 2(b), show the presence of several functional groups. The presence of −OH group stretching vibration is indicated by a peak at 3300 cm^−1^, possibly due to phenolic compounds such as rosmarinic acid, carnosic acid, and carnosol (Abdellatif et al. 2021). The presence of carbonyl groups (C=O), which may be attributable to flavonoids such as apigenin, luteolin, and kaempferol, is shown by the peak at 1640 cm^−1^ (Patil and Chougale 2021). The presence of aromatic compounds is indicated by the peak at 667 cm^−1^, which may be attributable to terpenoids such as eucalyptol, camphor, and α-pinene (Raj et al. 2018).

The Mn@NiO NC (Mn, Ni–O) stretching band corresponds to the peak at 418 cm^−1^ shown in Fig. 2(c). This peak confirms the successful biosynthesis of the Mn@NiO NC. A distinctive stretching band of the Mn@NiO NC indicates the strong interaction between Mn and Ni–O.

### Structure and morphology

SEM can provide detailed information about the shape and structure of NC. The SEM images of the Mn@NiO NC show foam-like shapes, suggesting a porous or spongy structure (Fig. 2e). This morphology can be advantageous for applications like catalysis, where a high surface area is desirable for increased reaction rates. The foam-like shape may have been achieved by using a templating agent during synthesis, which guides the growth of NC into specific shapes or structures. Alternatively, the self-assembly of Mn@NiO NC during synthesis, driven by various forces such as Van der Waals and electrostatic interactions, could have resulted in the foam-like morphology.

The particle size distribution histograms of the Mn@NiO NC are displayed in Fig. 2f. Through SEM data analysis, it was determined that the average particle size distribution for the Mn@NiO NC was approximately 36.7 nm.

### Zeta potential

The stability of colloidal systems, particularly nanoparticles in suspension, is a critical aspect influencing their performance and applicability in various fields. One crucial parameter in assessing this stability is the zeta potential, which provides insight into the surface charge of the particles. In the case of Mn@NiO NC synthesized from *Rosmarinus officinalis* L. extract, a zeta potential of −25.79 mV was observed (Fig. 2d), indicating a substantial negative charge on the particle surface. This high negative charge plays a pivotal role in preventing particle aggregation and clumping in the aqueous medium, thereby enhancing the overall stability of the NC. The use of *Rosmarinus officinalis* L. extract as a green synthesis method is noteworthy, as it not only contributes to the stability of the nanoparticles but also aligns with sustainable and environmentally friendly practices. The demonstrated ability to produce stable and well-dispersed nanoparticles through this method holds significant promise for diverse applications, underscoring the potential of botanical extracts in advancing nanomaterial synthesis for technological and biomedical purposes.

### BET analysis

**Fig. 3:**
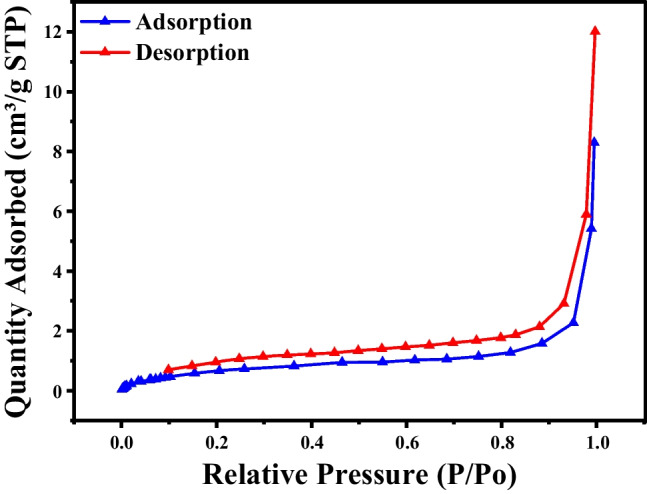
BET analysis graph depicting the N2 adsorption and desorption isotherms of Mn@NiO NC as a function of relative pressure

The nitrogen adsorption-desorption isotherms, as depicted in Fig. 3. offer an in-depth understanding of the porous structure and the surface area of the Mn@NiO NC. A surface area of 85.2 m^2^/g, calculated using the Brunauer–Emmett–Teller (BET) method, suggests a somewhat modest surface area. However, the material is identified as mesoporous, boasting an average pore diameter of 6.8 nm as per the Barrett-Joyner-Halenda (BJH) adsorption analysis and 5.3 nm according to BJH desorption analysis. The calculated pore volume, 0.21 cm^3^/g for adsorption and 0.19 cm^3^/g for desorption, is comparably low. The examination of the Mn@NiO NC surface using nitrogen adsorption-desorption isotherms unveils its mesoporous nature and potential catalytic activity. The presence of a type IV isotherm with a hysteresis loop affirms the existence of an ordered mesoporous framework texture. Although the specific surface area value is not notably high, the mesopores’ existence and the size of the nanoparticles could contribute to the Mn@NiO NC’s reported “enormous photocatalytic activity.” The Mn@NiO NC’s improved specific surface area value and superior nitrogen adsorption characteristics hint at its potential for high photocatalytic activity. The mesoporous structure, high surface area, and favorable pore characteristics of these nanoparticles make them beneficial for catalytic applications.

### Adsorption study of heavy metals

A series of adsorption experiments were conducted to evaluate the Mn@NiO NC capacity to remove heavy metals. For the various metals (Arsenic (III), Cadmium (II), Chromium (VI), Manganese (II), Molybdenum (II), Nickel (II), Lead (II), Antimony (III), Selenium (-II), and Zinc (II).), adsorption studies were conducted. A series of experiments were carried out to examine the loading capacity of eight different metal ions on a Mn@NiO NC. All heavy metals were used in the form of petroleum water to ensure that counter ions did not affect the behavior of the metals.

**Fig. 4:**
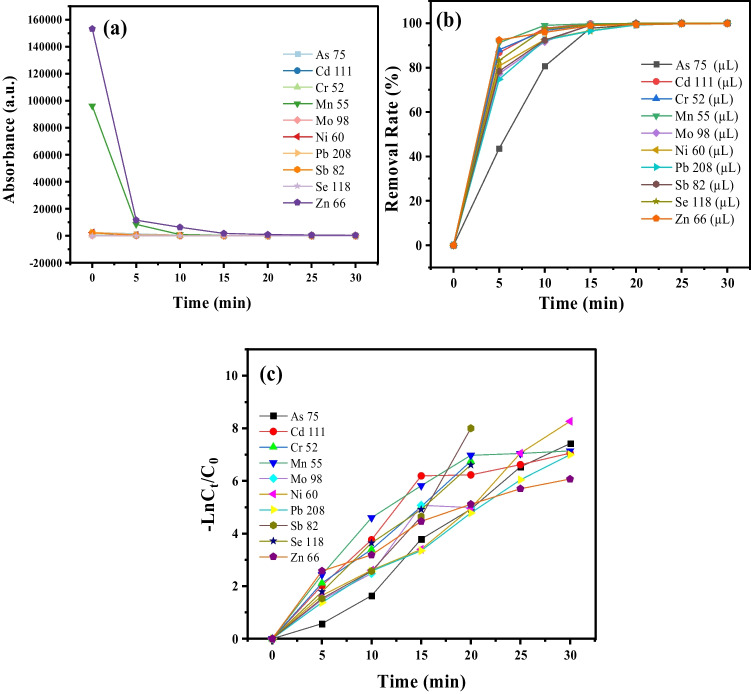
**a** Time effect and **b** efficiency rate of Mn@NiO NC for removal of heavy metals from the liquid phase. **c** First-order kinetics

The solution was then put through a 30-min process of sonication. The concentration of metal ions in the resulting supernatant was carefully measured using inductively coupled plasma mass spectrometry (ICPMS) after carefully collecting it. Fig. 4a shows the efficiency of heavy metal removal, which ranges from 99 to 100% for heavy metals.

A comprehensive investigation was carried out to elucidate the kinetics of adsorption for ten heavy metals, namely As, Cd, Cr, Mn, Mo, Ni, Pb, Sb, Se, and Zn. The relationship between the ln(*C*_*t*_/*C*_0_), and time, as illustrated in Fig. 4a. The results revealed that the adsorption process, catalyzed by the Mn@NiO NC, followed first-order reaction kinetics. Under optimized reaction conditions (Table 2), the kinetic parameters of adsorption were carefully examined. Graphical representations in Fig. 4b provided a comprehensive overview of the adsorption performance of biosynthetic Mn@NiO NC. The observed linearity between ln(*C*_*t*_/*C*_0_) and time in Fig. 4c confirmed adherence to first-order kinetics, as described by Eq. (6).

**Table 2:** Parameters of the first-order kinetic models for degradation of heavy metals by Mn@NiO NC

Model	Heavy metals	Fitting parameters	
First-order kinetic model		*k* (min^−1^)	*R*^2^
	As 75	0.26751 ± 0.01	0.984
	Cd 111	0.23443 ± 0.03	0.978
	Cr 52	0.32813 ± 0.01	0.995
	Mn 55	0.2358 ± 0.04	0.966
	Mo 98	0.27125 ± 0.04	0.937
	Ni 60	0.27113 ± 0.01	0.984
	Pb 208	0.23235 ± 0.005	0.996
	Sb 82	0.38222 ± 0.05	0.950
	Se 118	0.32675 ± 0.01	0.996
	Zn 66	0.18845 ± 0.02	0.913

### Photocatalytic performance of total suspended solids (TSS) and hydrocarbons from in oily water (OIW)

The objective of this study was to evaluate Mn@NiO NC photocatalytic efficiency in the degradation of hydrocarbons and total suspended solids (TSS) in oily water (OIW) at 25 °C. The results showed a decrease in the characteristic absorption shown in Table 3, demonstrating the successful degradation of TSS and hydrocarbons, as seen in Table 3, which highlights an improvement in the adsorption capacity with 30 min of contact time while maintaining a constant catalyst concentration, hydrocarbons, TSS content, and pH (Table 3).

**Table 3:** Photocatalytic parameters of hydrocarbon in oily water (OIW), suspended solids (TSS), and the chemical oxygen demand (COD)

	OIW (mg/L)	TSS (mg/L)	COD (mg/L)	pH
*t*_0_	540	148	6695	5.4
*t*=5 min	189.38	96.52	1845.76	5.8
*t*=10 min	61.17	41.05	938.18	6.1
*t*=20 min	23.49	23.61	482.49	6.3
*t*_*f*_= 30 min	9	18	103	6.5
Value limit (mg/L)	31	17	95	6.5–8.5
Removal %	98.3	87.3	98.4	-

The management of industrial wastewater containing a substantial amount of refractory chemical oxygen demand (COD) presents a significant challenge. Table 3 illustrates the industries responsible for releasing such wastewater with high refractory COD content. The conducted research in this study reveals that the NC technique exhibits exceptional efficiency in eliminating OWI, TSS, and COD from industrial wastewater, showcasing an impressive removal rate of 98.3%, 87.8%, and 98.4%, respectively, in 30 min under sunlight irradiation. Additionally, the Mn@NiO material demonstrates a remarkable capacity for effectively removing both oil and grease (OIW) as well as total suspended solids (TSS). Consequently, this novel composite (NC) exhibits considerable potential for implementation in the removal of OIW, TSS, and COD.

### Photocatalytic activity of organic dyes

**Fig. 5:**
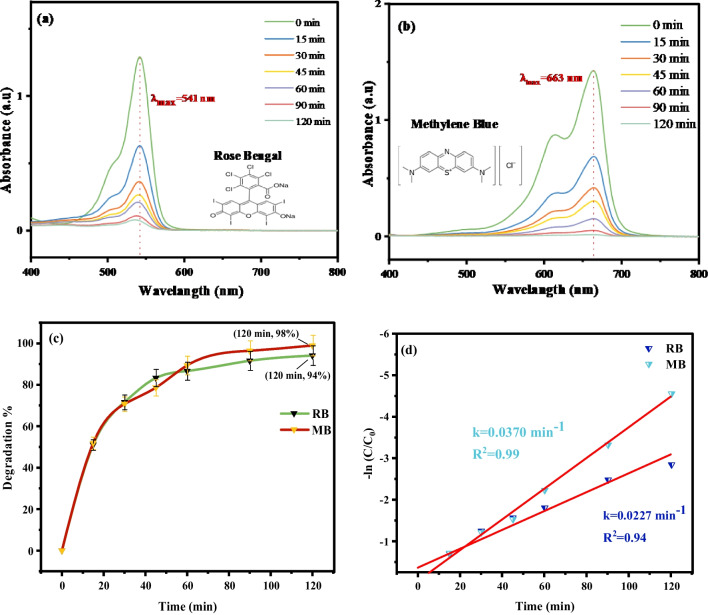
**a**, **b** The UV-visible spectra of Rose Bengal/RB and methylene blue/MB when exposed to sunlight in the presence of Mn@NiO NC are depicted. **c** Plot illustrating the degradation of dyes over time. **d** Kinetic plot showing the degradation of dyes by Mn@NiO NC

The research study examined the ability of biogenic Mn@NiO NC to act as a photocatalyst through a green approach. The effectiveness of the Mn@NiO NC was assessed by its capacity to break down RB and MB when exposed to sunlight irradiation. The findings in Fig. 5 indicated that the Mn@NiO NC demonstrated remarkable photocatalytic ability, displaying strong adsorption capabilities throughout the 120 min. Specifically, the Mn@NiO NC achieved a degradation rate of 94% for the RB dye solution (as shown in Fig. 5a, d), while the MB dye solution exhibited a degradation rate of 96.5% (Fig. 5c and d). The exceptional photocatalytic activity of the Mn@NiO NC can be attributed to its efficient recombination of (e^−^, h^+^) and generation of (−OH and O_2_^•−^), which play crucial roles in the process (Boutalbi et al. 2023).

The first-order kinetic model is frequently employed to characterize the kinetics of the photodegradation reaction for RB and MB. The equation derived from integrating this model is shown as (Eq. 6).

6 $$\ln \left(\frac{C}{C_0}\right)=-K\times t$$

The linearity of the Ln(*C*_*t*_/*C*_0_) progression, observed for all tested dyes, serves as a confirmation of the adherence to first-order kinetics. According to the Langmuir-Hinshelwood hypothesis, the photocatalysis process exhibits varying *K* values 0.0227 and 0.0370 min^−1^, for RB and MB dyes, respectively, as illustrated in Fig. 5(d). The kinetic rate constant of the photocatalytic reaction was determined using the Langmuir-Hinshelwood pseudo-first-order model. This was achieved by performing a linear fitting of ln(*C*_*t*_/*C*_0_) versus time, where the slope provided the kinetic rate constant (Ka). The degradation of RB and MB by Mn@NiO NC under sunlight exhibited rate constants of 0.0227 and 0.0370 min^−1^, respectively, Fig. 5(d). These results indicated a differential degradation rate between the dyes, with RB degrading at the fastest rate among MB. The inverse relationship between the rate constant values of the dyes and the degradation rate suggests that a decrease in the rate constant value corresponds to a decrease in the degradation rate. On the other hand, if the rate constant value increases, it indicates a higher efficiency in the degradation process. These results enhance our comprehension of the underlying mechanisms governing the photocatalytic degradation of diverse organic dyes, offering the potential for enhancing the overall degradation process efficiency.

**Fig. 6:**
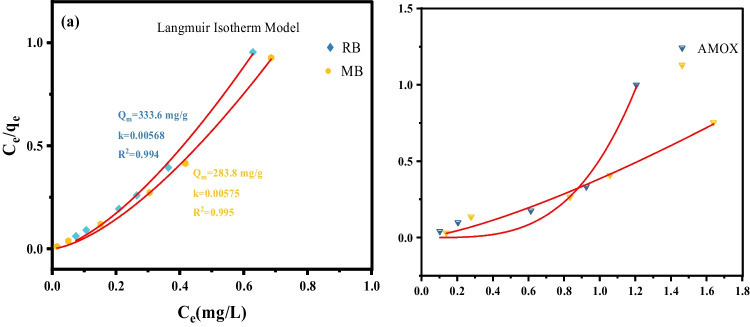
Langmuir isotherm model of **a** Rose Bengal and methylene blue. **b**. CEX and AMOX

The Langmuir isotherm model, which is commonly used to elucidate the adsorption mechanism and process of biogenic Mn@NiO NC, was applied in this study. Equation 7 represents the mathematical representation of the Langmuir isotherm model. The photocatalysis activity data, specifically the impact of initial Mn@NiO NC concentration on adsorption, was examined using this Langmuir isotherm model, as shown in Fig. 6. The regression (*R*^2^) values of 0.994 for RB and 0.995 RB for MB indicate a strong fit of the Mn@NiO NC degradation to the Langmuir model, proving the presence of a monolayer adsorption process (Table 4).

7 $$q={q}_m{k}_l{C}_e/1+{k}_l{C}_e$$

**Table 4:** Photocatalytic data of organic dyes and antibiotics

Dye and antibiotics	Degradation rate (%)	Constant rate (min^−1^)	Maximum adsorption capacity *Q*_*m*_ (mg/g)	Adsorption equilibrium constant (k)
Rose Bengal	94 ± 4.70	0.0227	333.6	0.00568
Methylene blue	98 ± 4.94	0.0370	283.8	0.00575
Cephalexin	97 ± 4.85	0.0619	387	0.00099
Amoxicillin	96 ± 4.8	0.0621	606	0.00084

### Antibiotic degradation activity

**Fig. 7:**
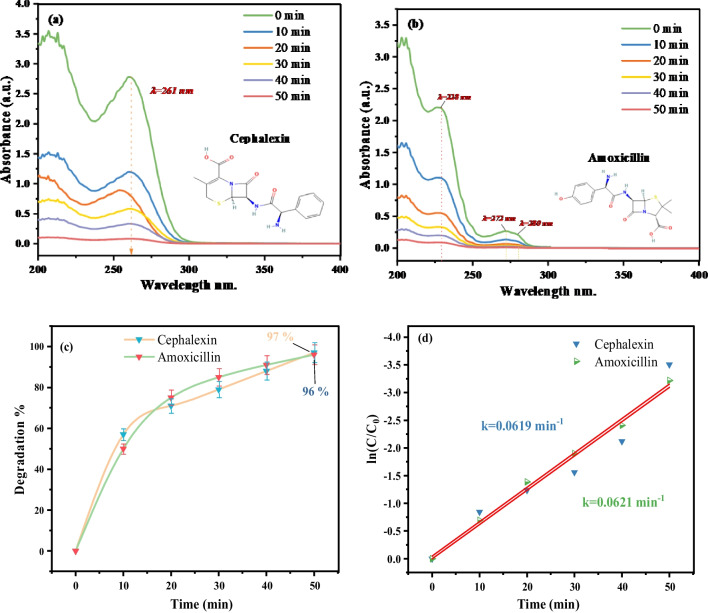
UV-Vis spectra of **a** cephalexin and **b** amoxicillin. **c** The degradation plots of antibiotics at 0.5 mg/10 ml. **d** Plot of the natural logarithm of the ratio of concentration to initial concentration (ln(*C*_*t*_/*C*_0_)) against time

The effectiveness of the biogenic Mn@NiO NC in promoting the removal of antibiotics, specifically CEX and AMOX, was assessed through a photodegradation reaction. This reaction involved the use of the catalyst Mn@NiO NC and NaOH. The results revealed that the Mn@NiO NC achieved a remarkable degradation rate, with 96% and 97% degradation of AMOX and CEX, respectively, attained within 50 min (Fig. 7a–d). The UV-Vis spectra of the reactions at various time intervals were analyzed to gain further insights. The findings indicated a gradual reduction in peak intensity at *λ*_max_ for CEX (261 nm) and AMOX (228 nm) as the exposure time increased. Eventually, these peaks disappeared entirely, indicating the completion of the degradation reactions (Fig. 7a, b). Moreover, by comparing the rate constants, it was determined that the degradation of AMOX exhibited a constant rate of *k* = 0.0621 min^−1^, which was slightly lower than the rate constant for CEX (*k* = 0.0619 min^−1^) as depicted in Fig. 7d. As a result, it is possible to conclude that the Mn@NiO NC exhibits good catalytic efficiency. This efficiency is ascribed to the synergistic effects of Mn and NiO nanoparticles, as well as the Mn@NiO NC large surface area.

Figure 7 d illustrates the concept of the Langmuir isotherm model, which proposes that when CEX and AMOX come into contact with the outer surface of Mn@NiO NC, they adhere to form a monolayer excluding any further stacking of the adsorbate. According to the Langmuir model, a state of equilibrium is achieved between the AMOX and CEX molecules that are adsorbed onto the Mn@NiO NC and the free CEX and AMOX ions present in the liquid phase (Table 4). This model suggests that the adsorption process reaches a point where the adsorption rate is equal to the desorption rate, establishing a dynamic balance between the adsorbate molecules on the NC surface and those in the surrounding liquid medium.

### Recycling performance

**Fig. 8:**
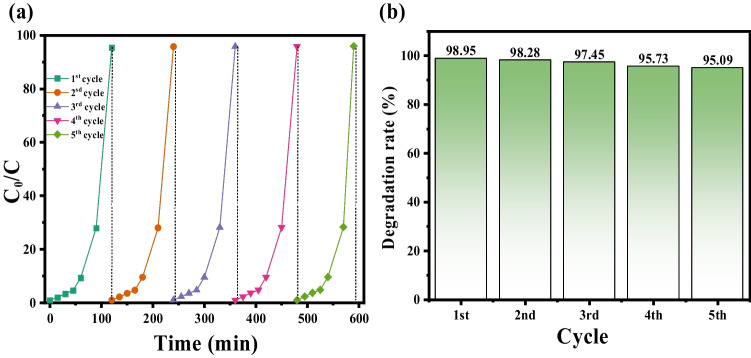
**a** Recyclability of the Mn@NiO photocatalyst for degradation of MB. **b** Degradation rate for the different cycles

The effectiveness of a photocatalyst in water purification processes depends significantly on its ability to be separated and reused. In assessing the reusability of the Mn@NiO catalyst, it was subjected to a drying process and then used in a second photocatalysis experiment, replicating the initial cycle’s conditions. The results of the photocatalyst’s recyclability over five consecutive cycles are presented in Fig. 8(a, b). The findings illustrate that the prepared Mn@NiO photocatalyst exhibited remarkable efficiency and reusability in degrading organic dyes or antibiotics. However, a slight reduction in photocatalytic activity was observed after five cycles, with a decrease from 99 to 97.8% for MB (Fig. 8b). This decline could be attributed to the loss of the catalyst during washing and centrifugation processes or the adsorption of certain intermediate species formed during photocatalysis.

**Fig. 9:**
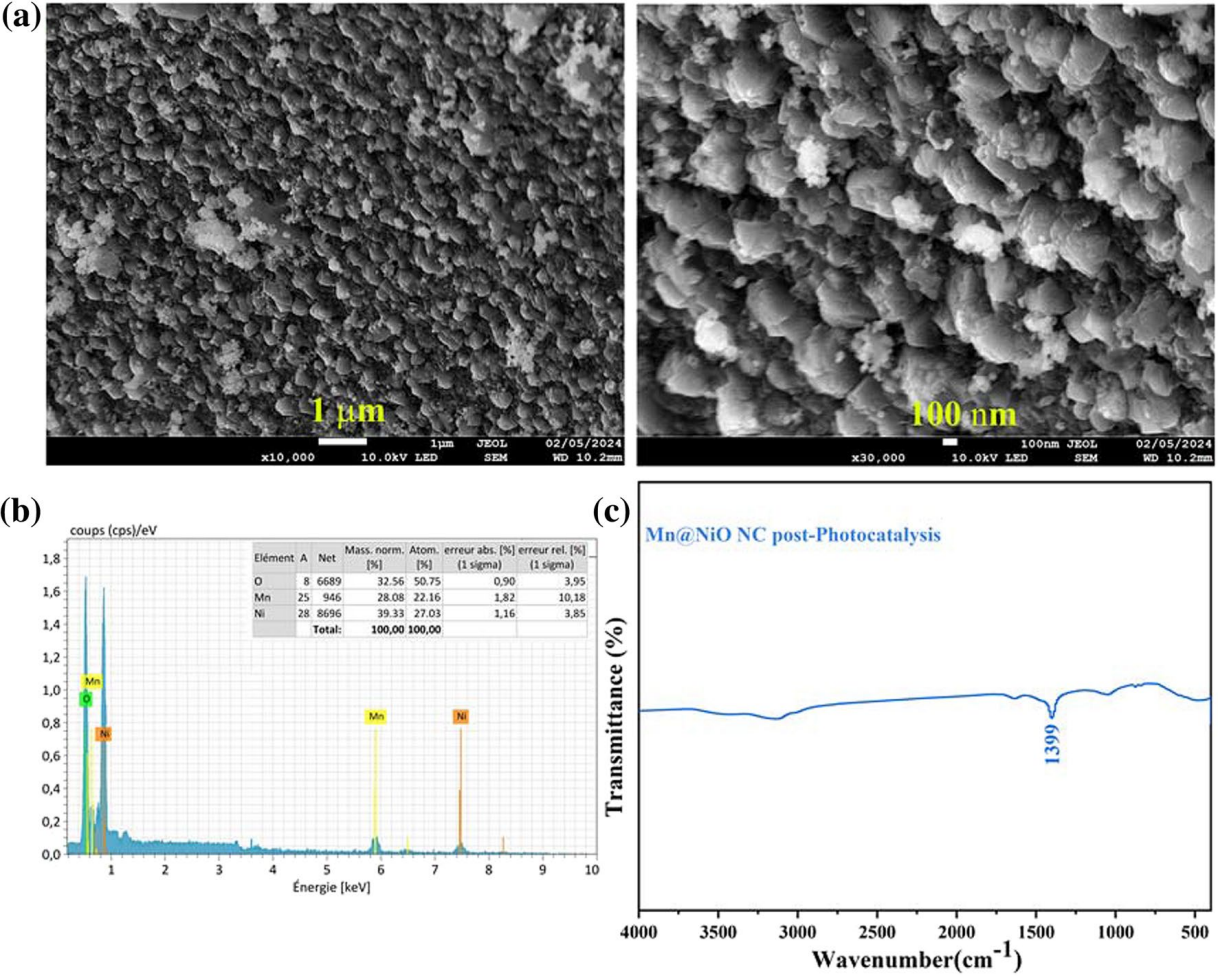
Mn@NiO NC characterization after photocatalytic reaction of MB. **a** SEM image. **b** EDX analysis. **c** FT-IR analysis

SEM analysis conducted after the fifth cycle of MB degradation reveals that the surface of Mn@NiO NC remains unaffected by the photocatalytic reaction. This indicates that the structural integrity and the surface morphology of the photocatalyst are stable, even after repeated cycles of photocatalytic activity. Furthermore, the EDX analysis confirmed the presence of Mn, Ni, and O without further elements (Fig. 9a, b). After the fifth cycle of the photocatalytic degradation of MB, FTIR spectroscopy revealed a peak (Fig. 9c). The peak at 1399 cm^−1^ is attributed to hydroxyl groups, which could be a trace from the dyes. This result shows that the chemical functional group of this NC is still with no change, which confirms the stability of the NC (El Gaayda et al. 2022), also indicates the successful degradation of MB without presenting any functional group.

### Photocatalytic mechanism

**Fig. 10:**
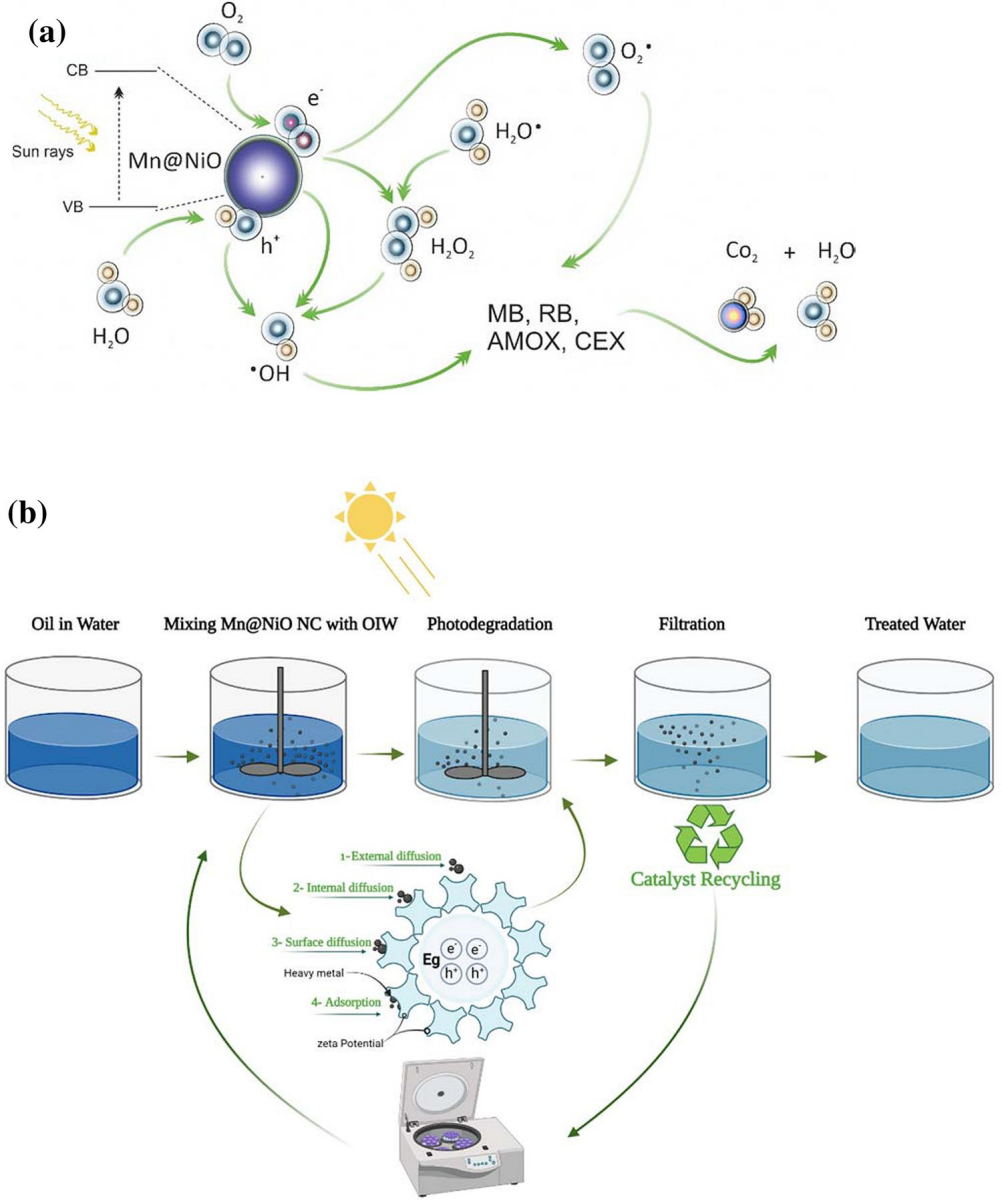
**a** Photocatalytic mechanism of Mn@NiO NC. **b** Experimental setup illustration and the mechanism adsorption site inside a particle

In order to explore the factors that contribute to the enhancement of the photocatalytic reaction of hydrocarbons, heavy metals, dyes, and antibiotics, Fig. 10a presents a proposed energy band diagram and charge transport mechanism for Mn@NiO NC. The photocatalytic reaction begins when the catalyst absorbs photons with higher energy than its bandgap energy. The excited electrons located at the valence band maximum (VB) are quickly promoted to the conduction band maximum (CB), initiating an oxygen reduction reaction. Simultaneously, the remaining holes at the VB can serve as oxidizing agents, driving oxidation reactions. This process generates active hydroxyl (•OH), superoxide (•O_2_ˉ), and other radicals that facilitate the degradation of hydrocarbons, heavy metals, dyes, and antibiotics. Additionally, the electrons from the CB of NiO can be transported to Mn (NPs), causing a downward band bending and efficient injection of excited electrons from NiO to Mn NPs. The accumulation of electrons on the surface of Mn NPs subsequently reacts with O_2_, resulting in the production of •O_2_^−^species. Deposition of Mn NPs onto the NiO surface not only enhances the ability to extract charges but also effectively inhibits the recombination of charge carriers, leading to an overall improvement in photocatalytic activity.

The enhanced photocatalytic activity of Mn@NiO NC can be attributed to its high surface area, which facilitates dye adsorption. Furthermore, the presence of Mn on the surface of NiO nanoparticles induces a localized surface plasmon resonance (SPR) effect. This effect enhances the absorption of visible light and reduces the recombination rate of electron-hole (e^−^ - h^+^) pairs. The degradation of organic dyes can be described by the following Eqs. (8–14) (Althamthami et al. 2023). Based on the surface charge of Mn@NiO, zeta potential analysis revealed a negative value of approximately −25.79 (Fig. 2d). This negativity plays a crucial role in decomposing positively charged organic pollutants. The strong electrostatic repulsion between individual molecules, facilitated by this negative charge, enhances the physical stability of nanosuspensions, thereby enhancing Mn@NiO’s ability to degrade pollutants. The generation of hydroxyl radicals is accountable for transporting solely unbound electrons, while superoxide anion radicals carry an additional negative charge. Considering the contrasting chemical characteristics of these two entities, it is logical to propose that the degradation mechanism commences by forming radicals, and this procedure should be initiated by producing hydroxylated radicals with minimal ion concentration in the solution Eqs. (8–10). Moreover, due to the inherent negative charge carried by anions, their interactions are anticipated to result in significant mineralization. This process is expected to occur with little to no presence of hydroxylated radicals and a plausible concentration of inorganic ions, assuming that any such ions exist within the organic framework. Following the generation of superoxide anions *O*^•^_2_ and hydroxyl radicals *OH*^•^ through the photocatalytic process, these species participate in reactions with organic compounds as depicted in the following equations (Bouafia et al. 2023).

8 $$\textrm{Mn}@\textrm{NiO}\overset{hv}{\to }\ {\textrm{e}}_{\left(C{B}^{-}\right)}+{h}_{\left(V{B}^{+}\right)}$$

Superoxide, an anion radical, is generated through a process of formation.

9 $${e}_{\left(C{B}^{-}\right)}+{\textrm{O}}_2\overset{\textrm{Mn}@\textrm{NiO}}{\to }{\textrm{O}}_2^{-}$$

The process of converting the OH^−^ group into OH through the action of the hole is known as neutralization.

10 $${h}_{\left(V{B}^{+}\right)}+{\textrm{H}}_2\textrm{O}\overset{\textrm{Mn}@\textrm{NiO}}{\to }{\textrm{H}}^{+}+\textrm{O}{\textrm{H}}^{-}$$

11 $${\textrm{H}}^{+}+{\textrm{O}}_2\to \textrm{H}{\textrm{O}}_2$$

12 $$\textrm{H}{\textrm{O}}_2+\textrm{H}{\textrm{O}}_2\to {\textrm{H}}_2{\textrm{O}}_2+{\textrm{O}}_2$$

13 $${\textrm{H}}_2{\textrm{O}}_2\overset{hv}{\to }2\textrm{HO}$$

14 $$\textrm{OIW},\textrm{TSS},\textrm{COD},\textrm{Dyes},\textrm{Antibiotics}+\textrm{HO}\ \overset{h^{+}}{\to}\textrm{C}{\textrm{O}}_2+{\textrm{H}}_2\textrm{O}$$

The adsorption process observed is intricate and involves multiple stages. Mn@NiO NC exhibits a substantial surface area due to their nanostructure, which provides numerous active sites conducive to adsorption. Heavy metals, oil and grease (OIW), total suspended solids (TSS), and chemical oxygen demand (COD) molecules adhere to the NC surface through various physical interactions, including Van der Waals forces, electrostatic attractions, and hydrogen bonding. Additionally, the metal oxide surfaces of Mn@NiO NCs can undergo ion exchange reactions, enabling heavy metal ions in water to replace metal ions on the NC surface through ion exchange processes, which is particularly effective for heavy metal removal. The functional groups on Mn@NiO NCs form complexes with heavy metal ions, OIW, TSS, and COD molecules through coordination bonds, covalent bonds, or electrostatic interactions, facilitating the adsorption of contaminants through surface complexation. Moreover, Mn@NiO NCs can promote chemical reactions that result in the elimination of impurities. Specifically, NiO, known for its catalytic properties, oxidizes organic compounds in OIW and COD, transforming them into less harmful substances. The porous structure of Mn@NiO NCs physically traps TSS particles and larger organic molecules in OIW and COD, immobilizing these impurities within the NC’s pores and effectively removing them from water or wastewater.

Figure 10b illustrates that understanding the parameters of adsorption equilibrium allows for determining the adsorption capacities of a given support. Additionally, predicting the adsorption curves requires determining the kinetic parameters. The process illustrated in Fig. 10b consists of several stages for transferring an adsorbate from the liquid phase to an adsorption site. Initially, there is an external diffusion phase wherein solute molecules migrate from the external liquid phase to the liquid phase bound to the solid particle, occurring through both diffusion and convection. Subsequently, an internal diffusion step occurs, where the solute moves within the liquid film toward the external surface of the adsorbent. The third stage involves the adsorbate diffusion within the adsorbent particle, driven by the concentration gradient. During this stage, the adsorbate molecule can diffuse either freely in the intraparticle liquid phase (referred to as migration on the surface, identifiable by the diffusion coefficient, Df) or in the adsorbed state, moving from one adsorption site to an adjacent one on the surface (termed migration on the surface, identifiable by the diffusion coefficient, Ds). Finally, the fourth stage encompasses the adsorption or fixation of the adsorbate onto the surface of the sorbent.

## Conclusion

This research explored the extraordinary potential of Mn@NiO NC synthesized through eco-friendly methods, aiming to eliminate heavy metals, hydrocarbons, total suspended solids (TSS), and chemical oxygen demand (COD) petroleum wastewater. The biosynthesis Mn@NiO NC exhibited outstanding capabilities in adsorption and photocatalytic degradation, providing a sustainable and effective solution for water purification and environmental remediation. The experimental findings indicated remarkably high efficiency in heavy metal removal, achieving percentages between 99 and 100% for diverse heavy metals such as As, Cd, Cr, Mn, Mo, Ni, Pb, Sb, Se, and Zn. Additionally, the NC demonstrated rapid removal of hydrocarbons, TSS, and COD, reaching rates of up to 98.3%, 87.8%, and 98.4%, respectively, within a brief 30 min. Furthermore, the Mn@NiO NC demonstrated significantly enhanced photocatalytic efficiency in eliminating RB, MB, CEF, and AMOX from aqueous solutions under sunlight irradiation, achieving impressive removal rates ranging from 90 to 100%. The kinetic analysis provided additional confirmation of the NC’s advantageous adsorption capacity for a diverse range of contaminants.

### Supplementary information

ESM 1 (DOCX 29 kb)

## Data Availability

The data discussed in the manuscript are included in the text itself.
