# Supplementary material for: Nanostructured Mn@NiO composite for addressing multi-pollutant challenges in petroleum-contaminated water
Source: Environ Sci Pollut Res Int. 2024 Jun 28;31(31):44254–71. doi: 10.1007/s11356-024-34012-3 (PMC11252200; doi:10.1007/s11356-024-34012-3)
Supplement: Supplementary file 1 — (DOCX 29 kb) [file 11356_2024_34012_MOESM1_ESM.docx]

*Supplementary information for*

**Nanostructured Mn@NiO Composite for Addressing Multi-Pollutant Challenges in Petroleum-Contaminated Water**

**Gamil Gamal Hasan^1*^, Salah Eddine Laouini^2^, Ahmed I. Osman^3*^, Abderrhmane Bouafia^2^, Mohammed Althamthami^4^, Souhaila Meneceur^2^, Iman kir^2^, Hamdi Mohammed^2^, Brock Lumbers^5^, David W. Rooney^3^**

^1^Laboratory of Valorisation and Technology of Sahara Resources (VTRS), El Oued University, El Oued, 39000, Algeria.

^2^Laboratory of Biotechnology Biomaterials and Condensed Matter, Faculty of Technology, University of El Oued, El Oued 39000, Algeria.

^3^ School of Chemistry and Chemical Engineering, Queen's University Belfast, Belfast BT9 5AG, Northern Ireland, UK.

^4^Physics Laboratory of Thin Films and Applications, Biskra University, BP 145 RP, Biskra 07000, Algeria

^5^Faculty of Technology and Bionics, Rhine-Waal University of Applied Sciences, Marie-Curie-Straße 1, 47533 Kleve, Germany (brock.lumbers@hsrw.org)

*Corresponding Author: Ahmed I. Osman; Email: [aosmanahmed01@qub.ac.uk](mailto:aosmanahmed01@qub.ac.uk)

Address: School of Chemistry and Chemical Engineering, Queen's University Belfast, David Keir Building, Stranmillis Road, Belfast BT9 5AG, Northern Ireland, United Kingdom

Fax: +44 2890 97 4687

Tel.: +44 2890 97 4412

**Inductively Coupled Plasma Mass Spectrometry (ICP-MS)**

is a highly sensitive technique for quantifying metal proportions. It enables the concurrent detection of various ions within a single solution. The ICP-MS product from HTDS, the NexION 2000 model, primarily comprises a water-cooled spray chamber, a gas mass flow controller for nebulizer gas, plasma gas, and auxiliary gases, and a nebulizer equipped with a variable speed peristaltic pump. It also includes a valve system with a 0.5 mL injection loop, an impurity extractor, and an automatic sampler with an injection system. The system operates on Syngetix software and uses 20 mL polypropylene conical tubes with polypropylene caps.

The system requires argon and helium gas supply cylinders with 99.9999% purity each, ultra-pure water, a multi-element stock standard solution with each element at a concentration of 1000 mg/litre, mono-elemental standard solutions, and solutions of the reference element (internal standard) or optimization solution for both the standard mode and the Kinetic Energy Discrimination (KED) mode.

Adsorbents such as particles of Manganese (Mn) and Nickel Oxide (NiO) are synthesized using the green method.

**The method developments for quantifying water samples after the adsorption study:**

• After the adsorption study, metal ion concentrations in the liquid supernatant were determined using ICP-MS analysis.

• Method development involved calibration of the instrument with standard solutions containing known concentrations of metal ions.

• Sample preparation included appropriate dilution and matrix matching to ensure accurate quantification.

• Parameters such as nebulizer gas flow rate, RF power, and dwell time were optimized for sensitivity and precision.

• Data analysis was performed using Syngetix operating software, including linear regression analysis and adjustment with internal standards.

**2. TD-172 500 Hydrocarbon Analyzer (Turner Designs Hydrocarbon Instruments):**

The TD-172 500D Oil in Water Meter, an analyzer (Revision: C, P/N 100,668) from the United States, employs UV fluorescence to ascertain the oil content in oily water, which may contain crude oil or gas condensates. An oil in water analyzer (TD-172 500, Turner Designs Hydrocarbon Instruments, USA) is utilized to quantify the amount of oil or Oil in Water (OIW) in the produced or separated water.

**The method developments for quantifying water samples:**

Post-treatment of oily water samples from the photocatalysis study involved quantification of hydrocarbons using the TD-172 500 meter.

• Method development included calibration of the instrument with standardized samples containing known oil content.

• Sample preparation involved extraction of hydrocarbons using hexane and subsequent analysis using the TD-172 500 meter.

• Parameters such as sample volume, solvent volume, and shaking duration were optimized for efficient extraction.

• The response value provided by the analyzer was recorded and correlated with hydrocarbon concentration in parts per million (ppm).

**3. UV–Visible spectrophotometer**

In the field of water analysis, suspended solids (SS) are all undissolved substances that are perceptible to the unaided eye and are present in the water. These substances can be examined using a UV–visible spectrophotometer, specifically the mat-lib DR6000 model. A typical test involves a 10 mL sample of petroleum water. This oily water is a byproduct from the separation processes of crude oil extraction in the HASSI MESSAOUD (31.201183,5.740473), Ouargla, Algeria.

**The method developments for quantifying water samples:**

• For the quantification of Total Suspended Solids (TSS) after the photocatalysis study, UV-visible spectrophotometry was employed.

• Method development included calibration of the instrument with standardized samples containing known TSS concentrations.

• Sample preparation involved transferring a precise volume of water sample containing petroleum into a transparent vessel for analysis.

• The vessel was then inserted into the UV-Visible spectrophotometer for analysis.

• Parameters such as wavelength, path length, and integration time were optimized for accurate measurement.

• The resulting measurement was observed and displayed on the instrument's screen for recording.
